# The splicing factor SRSF6 mediates ferroptosis resistance in head and neck squamous cell carcinoma through induction of stearoyl-CoA desaturase

**DOI:** 10.1016/j.jbc.2025.110509

**Published:** 2025-07-23

**Authors:** Lingyan Yan, Jiayun Wang, Bingrong Li, Jihua Guo, Rong Jia

**Affiliations:** State Key Laboratory of Oral & Maxillofacial Reconstruction and Regeneration, Key Laboratory of Oral Biomedicine Ministry of Education, Hubei Key Laboratory of Stomatology, School & Hospital of Stomatology, Wuhan University, Wuhan, Hubei, China

**Keywords:** ferroptosis, SRSF6, FTO, m6A, head and neck squamous cell carcinoma (HNSC)

## Abstract

Ferroptosis has become a promising target for cancer treatment since its discovery. However, tumor cells reinforce the defense systems to strengthen ferroptosis resistance. Here, we reported a positive feedback loop containing the serine and arginine rich splicing factor 6 (SRSF6) and fat mass and obesity-associated protein (FTO), which played crucial roles in the enhancement of cellular ferroptosis resistance and tumor progression in head and neck squamous cell carcinoma. Mechanistically, the SRSF6/FTO loop activated the sterol regulatory element binding transcription factors signaling pathway through FTO-mediated m6A demethylation, especially the upregulation of stearoyl-CoA desaturase, a key defender against ferroptosis. These findings provided new insights and directions for understanding the ferroptosis resistance of tumor cells.

Ferroptosis, an iron-dependent form of cell death characterized by the lethal accumulation of reactive oxygen species (ROS) and excessive lipid peroxidation, has attracted considerable attention in tumor progression and antitumor therapy (1, 2, 3). Mounting evidence has proved that triggering ferroptosis is a promising treatment modality for tumors, and several ferroptosis inducers targeting ferroptosis-related genes have shown antitumor effects (4). However, some tumors have been identified to develop the reinforcement of cellular defense systems against ferroptosis, such as strengthening the antioxidant defense systems or promoting the synthesis of monounsaturated fatty acid-containing phospholipid (MUFA-PL) that inhibits lipid peroxidation, through altering the intrinsic gene expression patterns (5). Therefore, the exploration of the underlying mechanisms by which cells resist ferroptosis has garnered increasing attention.

The serine and arginine rich splicing factor (SRSF) family, which recognizes and binds RNA through their RNA recognition motifs, is key in regulating the fate of pre-mRNA through processes such as alternative splicing (AS) (6, 7), mRNA transport, and nonsense-mediated mRNA decay (8). Abnormally elevated SRSF expression, accompanied by aberrant splicing events, has been identified in multiple cancers such as breast cancer and head and neck cancer (9, 10). However, the role of SRSF proteins in ferroptosis and corresponding antitumor therapy remains largely unclear. SRSF6 has been reported as a proto-oncogene that is overexpressed in human skin cancer (11) and colorectal cancer (12). Moreover, SRSF6 has been shown to promote hyperplasia in sensitized skin (11) and enhance the cell proliferation, migration, and invasion in colorectal (12). Nevertheless, the reasons for the elevated level of SRSF6 in cancer tissues and its role in ferroptosis are still unclear.

As the most abundant mRNA modification in eukaryotes, N6-methyladenosine (m6A) plays a crucial role in the occurrence and progression of tumors. Fat mass and obesity-associated protein (FTO) is the first identified RNA m6A demethylase, capable of removing the m6A modifications at the “RRACH” (R: A/G; H: A/C/U) sites on mRNAs and long noncoding RNAs (13). FTO deficiency has been reported to sensitize colorectal cells to ferroptosis by inhibiting the expression of solute carrier family 7 member 11 (SLC7A11) or glutathione peroxidase 4 (GPX4) (14). However, the role of FTO, particularly its m6A demethylase function, in ferroptosis resistance was still largely unclear. Although FTO has been identified as being highly expressed in various tumors and closely related to tumor progression (15, 16), the molecular mechanism accounting for the upregulation of FTO in tumor tissues remains largely unknown. Furthermore, the roles and the underlying mechanism of FTO as well as m6A modification in tumor progression, which might provide new ideas for antitumor therapy, deserve further investigation.

In this study, we reported a positive feedback loop between SRSF6 and FTO, which helped head and neck squamous cell carcinoma (HNSC) cells resist ferroptosis and promoted HNSC progression. Firstly, we revealed the expression profiles of SRSF6 and FTO in HNSC. Subsequently, we analyzed the effects of SRSF6 and FTO on cell proliferation and ferroptosis. Mechanistically, the specific mutual regulatory mechanism underlying the positive feedback loop containing SRSF6 and FTO was analyzed. Following this, we explored the common downstream target genes and signaling pathways. Therefore, our work highlighted the crucial roles of the SRSF6/FTO feedback loop in tumor cell ferroptosis resistance and tumor progression, which provided potential therapeutic targets for antitumor treatment.

## Results

### SRSF6 confers cellular ferroptosis resistance in HNSC

**Figure 1:**
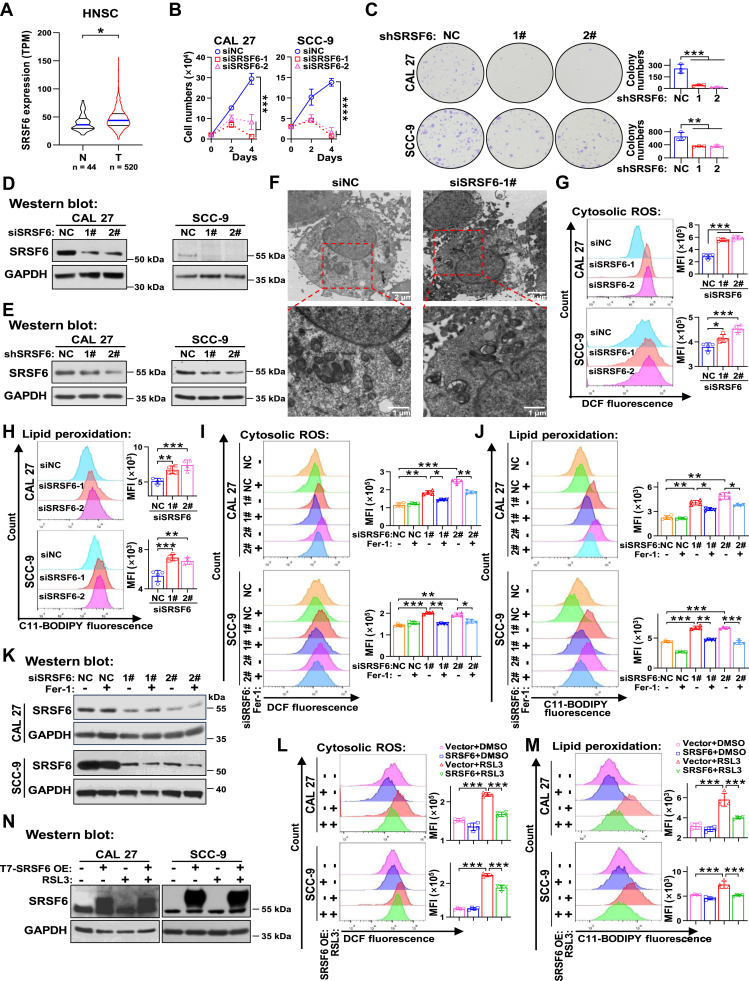
**SRSF6 confers resistance to ferroptosis in head and neck squamous cell carcinoma (HNSC) cells.***A*, the statistical analysis of the transcriptional level of SRSF6 in HNSC tissues and normal tissues from the TCGA database. The gene expression data were downloaded from the oncoDB website. *B*, proliferation curves of CAL 27 and SCC-9 (two head and neck squamous cell carcinoma cells) transfected with specific siRNAs targeting SRSF6 (siSRSF6-1#, siSRSF6-2#) and negative control siRNA (siNC). Data are means ± SD, n = 4. *C*, colony formation assays in CAL 27 and SCC-9 cells after SRSF6 stably silenced by specific shRNAs (shSRSF6-1#, shSRSF6-2#). Negative control shRNA (shNC) was used as a control. The colonies with more than 50 cells were counted. Data are means ± SD, n = 3. *D* and *E*, knockdown efficiency of siSRSF6 (*D*) and shSRSF6 (*E*) in CAL 27 and SCC-9 cells was confirmed by Western blot and GAPDH served as a loading control. *F*, representative TEM images of mitochondria in CAL 27 cells transfected with siSRSF6-1# and siNC. *G*–*N,* cytosolic ROS production was detected using the fluorescent probe DCF. Lipid peroxidation was analyzed using the C11-BODIPY (581/591) probe (lipid peroxidation sensor). *G* and *H*, flow cytometry analysis of cytosolic ROS (*G*) and lipid peroxidation (*H*) after CAL 27 and SCC-9 cells transfected with siSRSF6-1# and siSRSF6-2#. siNC served as a negative control. *I*–*K*, after transfected with siRNAs, CAL 27 and SCC-9 cells were treated with the antioxidant ferrostain-1 (Fer-1) and DMSO. The levels of ROS (*I*) and lipid peroxidation (*J*) were analyzed by flow cytometry. The knockdown efficiency (*K*) was analyzed by Western blot. *L*–*N*, CAL 27 and SCC-9 cells with or without stable SRSF6 overexpression were treated with a ferroptosis inducer, RAS-selective lethal 3 (RSL3). Flow cytometry was used to analyze the levels of ROS (*L*) and lipid peroxidation (*M*). The representative picture of flow cytometry is shown on the left and the mean fluorescence intensity (MFI) measured is on the right. Western blot was used to detect the overexpression of T7-SRSF6 (*N*). Data are means ± SD, n = 3 or 4. ∗*p* < 0.05, ∗∗*p* < 0.01, ∗∗∗*p* < 0.001, ∗∗∗∗*p* < 0.0001. DMSO, dimethyl sulfoxide; ROS, reactive oxygen species; SRSF, serine and arginine rich splicing factor; TCGA, The Cancer Genome Atlas; TEM, transmission electron microscope.

To characterize the role of SRSF6 in tumor progression, we first analyzed the transcriptional level of SRSF6 in normal and tumor tissues from The Cancer Genome Atlas (TCGA) database. SRSF6 mRNA level was significantly elevated in tumor tissues in various cancers including HNSC (Figs. 1*A* and S1, *A*–*H*). Furthermore, SRSF6 was positively correlated with two established tumor cell proliferation markers, Ki67 (r = 0.2862, *p* < 0.0001, n = 564, Fig. S1*I*) and proliferating cell nuclear antigen (PCNA; r = 0.5146, *p* < 0.0001, n = 564, Fig. S1*J*) in HNSC tissues, including tumor and normal tissues, from the TCGA database. Consequently, we proceeded to evaluate the effects of SRSF6 on HNSC cell proliferation. Knockdown experiments demonstrated that SRSF6 silencing significantly inhibited cell proliferation and colony formation in CAL 27 and SCC-9 cells (Fig. 1, *B*–*E*).

To elucidate the reasons for the observed decrease in proliferation, we next investigated morphological changes at the ultrastructural level using transmission electron microscope. SRSF6 depletion led to mitochondrial shrinkage and the disappearance of mitochondrial cristae structures, both of which are characteristic morphological features of ferroptosis, along with the engulfment of several damaged mitochondria (Fig. 1*F*). Ferroptosis is an iron-dependent form of cell death, the key features of which are the accumulation of ROS and excessive lipid peroxidation of cellular membranes. As expected, the knockdown of SRSF6 significantly enhanced the cellular ROS generation (Fig. 1*G*) and lipid peroxidation (Fig. 1*H*) in CAL 27 and SCC-9 cells. Ferrostatin-1 (Fer-1), a strong antioxidant and specific inhibitor of ferroptosis (17), effectively mitigated the cellular proliferation inhibition (Fig. S2), the overproduction of ROS (Fig. 1*I*) and lipid peroxidation (Fig. 1*J*) induced by SRSF6 silencing (Fig. 1*K*) in CAL 27 and SCC-9 cells.

Building on these observations, we sought to further investigate the interplay between SRSF6 expression and the susceptibility of HNSC cells to ferroptosis. RAS-selective lethal 3 (RSL3) is a well-known ferroptosis agonist (17), which also induced the overgeneration of ROS (Fig. 1*L*) and cellular lipid peroxidation (Fig. 1*M*) in HNSC cells. Notably, the overexpression of SRSF6 (Fig. 1*N*) significantly counteracted ferroptosis triggered by RSL3, as evidenced by the reduction in ROS levels (Fig. 1*L*) and lipid peroxidation (Fig. 1*M*). Collectively, these results suggest that SRSF6 ablation induces ferroptosis, while overexpressed SRSF6 confers cellular resistance to ferroptosis in HNSC cells. In addition, conserved ferroptosis resistance upon SRSF6 overexpression was observed in nontransformed human epithelial cells (HaCaT, Fig. S3), suggesting the biological importance of SRSF6 in regulating ferroptosis.

### SRSF6 depletion retards tumor growth through inhibiting FTO expression

**Figure 2:**
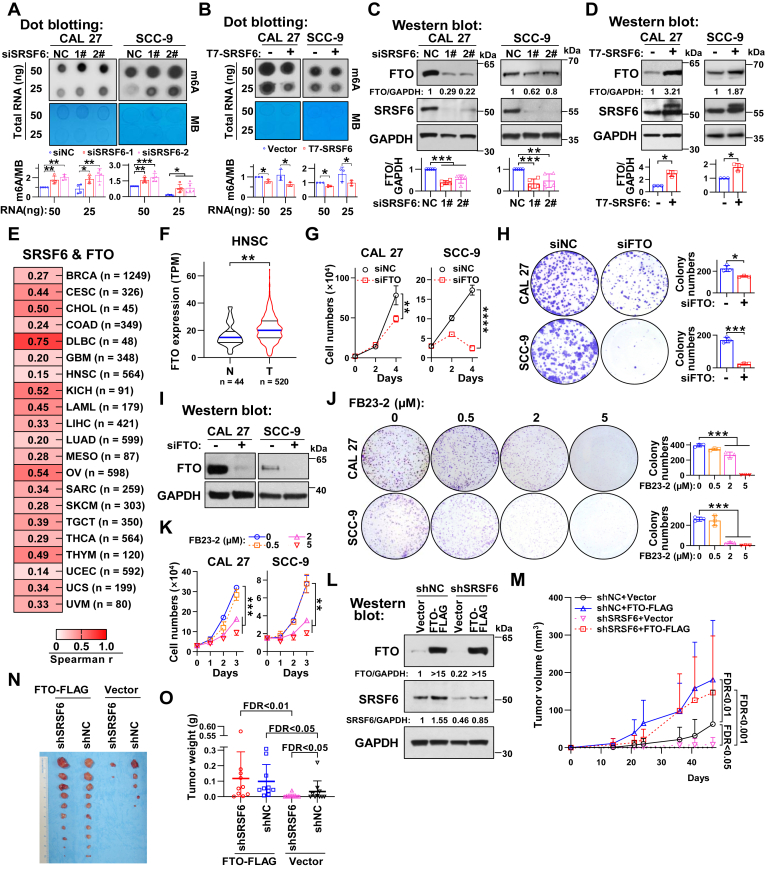
**SRSF6 depletion inhibits tumor growth through the ablation of FTO expression in HNSC.***A* and *B*, dot blot assay analyzed the total RNA m6A abundance of CAL 27 or SCC-9 cells with knockdown of SRSF6 (*A*) or with forced overexpression of SRSF6 (*B*). siNC (*A*) and empty vector (*B*) were used as negative controls, respectively. Methylene blue (MB) staining served as a loading control. The histograms below summarize the statistical results of the dot blot assay. *C* and *D*, Western blot analysis of FTO and SRSF6 after CAL 27 or SCC-9 cells silencing (*C*) or overexpressing (*D*) SRSF6. siNC (*C*) and empty vector (*D*) were used as negative controls, respectively. GAPDH served as a loading control. The histograms below summarize the relative expression levels of FTO. *E* and *F*, the analysis of mRNA levels of FTO and SRSF6 in tumor tissues and normal tissues from the TCGA database. The gene expression data were downloaded from the oncoDB website. *E*, Spearman correlation between FTO and SRSF6 in cancers (including tumor tissues and normal tissues, Spearman r > 0.1, *p* < 0.05). *F*, statistical analysis of FTO mRNA expression in tumor tissues and normal tissues in HNSC. *G*–*I*, CAL 27 and SCC-9 cells were transfected with specific siRNA targeting FTO (siFTO) and negative control siRNA (siNC). *G*, cell proliferation curves. *H*, cell colony formation analysis. The colonies with more than 50 cells were counted. *I*, verification of FTO knockdown by siFTO using Western blot. GAPDH served as a loading control. *J* and *K*, CAL 27 and SCC-9 cells were treated with 0, 0.5, 2, and 5 μM FTO inhibitor FB23-2. *J*, cell colony formation analysis. Cell clusters with more than 50 cells were counted as clones. *K*, cell proliferation curves. *L*–*O*, CAL 27 cells stably expressing shSRSF6 or shNC were stably cotransfected with FTO-FLAG or empty vector control plasmids. Then the cells were injected into nude mice subcutaneously. *L*, Western blot analyzed the expression of FTO and SRSF6 in CAL 27 cells. GAPDH served as a loading control. *M*, growth curve of tumors. Tumor volume was calculated as (length × width^2^ × π/6). *N* and *O*, the mice were sacrificed at day 47, and subcutaneous tumors were collected, weighed, and photographed immediately. Data are means ± SD. ∗*p* < 0.05, ∗∗*p* < 0.01, ∗∗∗*p* < 0.001, ∗∗∗∗*p* < 0.0001. FTO, fat mass and obesity-associated protein; HNSC, head and neck squamous cell carcinoma; m6A, N6-methyladenosine; SRSF, serine and arginine rich splicing factor; TCGA, The Cancer Genome Atlas.

m6A is the most prevalent RNA modification in eukaryotic species (18). Previous studies have shown that alterations in RNA m6A modification are strongly associated with programmed cell death including ferroptosis (14, 19). In the present study, SRSF6 silencing significantly increased the m6A modification level of total RNA in CAL 27 and SCC-9 cells (Fig. 2*A*). Conversely, SRSF6 overexpression resulted in a significant decrease in the levels of total RNA m6A modification (Fig. 2*B*). Furthermore, the protein levels of FTO, an m6A demethylase, were significantly decreased (Fig. 2*C*) or increased (Fig. 2*D*) upon the deletion or overexpression of SRSF6 in HNSC cells, respectively.

To further investigate the mechanism by which SRSF6 regulates FTO, considering SRSF6’s role as a splicing factor, we first examined the regulatory effect of altered SRSF6 expression on the potential AS of FTO exons 2 to 8 shown in the NCBI gene database. The results revealed that neither overexpression nor knockdown of SRSF6 affected the AS of exons 2 to 8 (Fig. S4*A*). Then we conducted RNA immunoprecipitation (RIP) assays using the anti-T7 antibody in cells transfected with T7-SRSF6 expression plasmid, and found the physical interaction between SRSF6 protein and FTO mRNA (Fig. S4*B*). Further analysis of the NCBI gene database indicated that FTO mRNA exhibits alternative polyadenylation, which generates two types of FTO transcripts with long (10 kb) or short (2.5 kb) 3′ UTR (Fig. S4*C*). Then we analyzed the expression levels of these two types of transcripts in CAL 27 and SCC-9 cells with altered SRSF6 expression. The results indicated that SRSF6 depletion significantly enhanced distal polyadenylation (polyA) site selection, producing FTO mRNA with long 3′ UTR (Fig. S4*D*). In contrast, SRSF6 overexpression markedly inhibited the production of FTO mRNA with long 3′ UTR (Fig. S4*E*). To functionally characterize these 3′ UTR variants, we engineered GFP reporter constructs harboring either the proximal (short) or distal (long) polyadenylation signal-derived 3′ UTR sequences (Fig. S4*F*). After transfected into cells, the long 3′ UTR inhibited GFP protein expression compared with the short 3′ UTR (Fig. S4*G*). These results indicated that SRSF6 promoted FTO protein expression through polyadenylation signal selection-mediated 3′ UTR shortening.

To delve deeper into the role of FTO in HNSC progression and its functional interplay with SRSF6, we examined its expression profile. A significant positive correlation was observed between the transcriptional levels of FTO and SRSF6 across multiple cancer types (including tumor and normal tissues) from the TCGA database (Fig. 2*E*). Corresponding with SRSF6, FTO was also found to be overexpressed in tumor tissues in HNSC (Fig. 2*F*). These results suggest the pivotal role of SRSF6 in promoting FTO protein expression.

To delineate the functional role of FTO in HNSC progression, we evaluated its impact on cell proliferation and colony formation. Our findings revealed that FTO silencing significantly impeded the proliferation of HNSC cells (Fig. 2*G*) and their ability to form colonies (Fig. 2, *H* and *I*). Subsequently, to verify that the effect of FTO on cell proliferation is related to its demethylase function, we employed FB23-2, a specific FTO inhibitor known to target the m6A demethylase function of FTO by directly binding to the substrate-binding pocket of the FTO protein (20). Treatment with FB23-2 significantly suppressed colony formation (Fig. 2*J*) and slowed down cell proliferation (Fig. 2*K*) in a dose-dependent manner in CAL 27 and SCC-9 cells. To further elucidate the collective influence of the SRSF6-FTO axis on HNSC progression, we conducted a tumorigenesis assay in nude mice *in vivo*. Notably, the deletion of SRSF6 effectively hindered tumor growth (Fig. 2, *L*–*O*, shNC + Vector & shSRSF6 + Vector, false discovery rate (FDR) < 0.05). Moreover, the overexpression of FTO not only significantly promoted tumor growth (Fig. 2, *L*–*O*, shNC + Vector & shNC + FTO-FLAG, FDR < 0.05), but also reversed the growth inhibition induced by shSRSF6 (Fig. 2, *L*–*O*, shSRSF6 + Vector & shSRSF6 + FTO-FLAG, FDR < 0.01). These results demonstrate that SRSF6 depletion inhibits tumor growth through the ablation of FTO expression in HNSC.

### FTO confers ferroptosis resistance in HNSC cells

**Figure 3:**
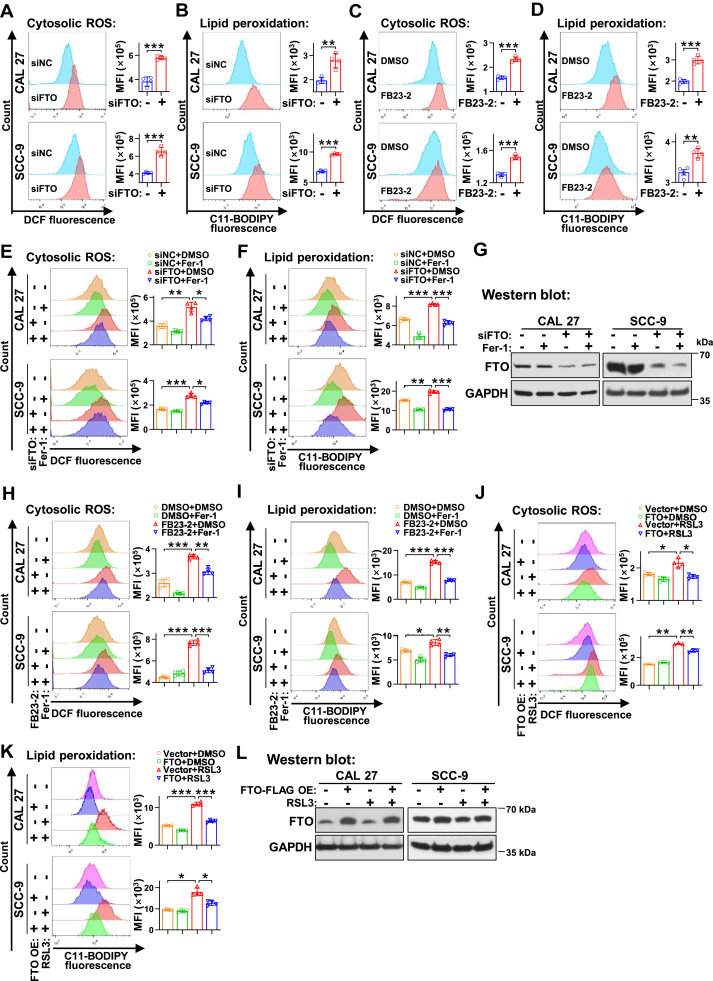
**FTO promotes ferroptosis resistance in HNSC cells.***A* and *B*, analysis of cytosolic ROS generation (*A*) or lipid peroxidation (*B*) by flow cytometry after CAL 27 and SCC-9 cells transfected with siFTO or siNC. *C* and *D*, measurement of cytosolic ROS generation (*C*) and lipid peroxidation (*D*) by flow cytometry after CAL 27 and SCC-9 cells treated with FB23-2 or DMSO. *E*–*I*, after transfected with siFTO and siNC (*E*–*G*), or treated with FB23-2 and DMSO (*H* and *I*), CAL 27 and SCC-9 cells were treated with Fer-1 and DMSO. Flow cytometry was used to detect the cytosolic ROS (*E* and *H*) and lipid peroxidation (*F* and *I*). Western blot was used to detect the knockdown efficiency of siFTO and GAPDH served as a loading control (*G*). *J*–*L*, CAL 27 and SCC-9 cells stably overexpressing FTO or empty control vector were treated with RSL3 or DMSO. Flow cytometry was used to analyze the cytosolic ROS (*J*) and lipid peroxidation (*K*). Western blot was used to analyze the overexpression of FTO-FLAG (*L*). Data are means ± SD, n = 3 or 4. ∗*p* < 0.05, ∗∗*p* < 0.01, ∗∗∗*p* < 0.001. DMSO, dimethyl sulfoxide; Fer-1, ferrostatin-1; FTO, fat mass and obesity-associated protein; HNSC, head and neck squamous cell carcinoma; ROS, reactive oxygen species; RSL3, RAS-selective lethal 3.

To determine the role of FTO in ferroptosis, we measured the ROS production and the extent of lipid peroxidation in cells following siFTO transfection or FB23-2 treatment. Notably, the silencing of FTO induced a pronounced enhancement in ROS generation (Fig. 3*A*) and lipid peroxidation (Fig. 3*B*) in CAL 27 and SCC-9 cells. Meanwhile, the suppression of FTO’s m6A demethylase activity by FB23-2 resulted in a significant elevation of ROS level (Fig. 3*C*) and enhanced lipid peroxidation (Fig. 3*D*) in HNSC cells. Moreover, the ferroptosis inhibitor Fer-1 could rescue the ferroptosis induced by FTO silencing (Figs. 3, *E*–*G* and S5) in CAL 27 and SCC-9 cells, as evidenced by the mitigation of proliferation inhibition, cellular ROS generation, and lipid peroxidation. Similarly, the ferroptosis induced by FB23-2 could be rescued by Fer-1 (Fig. 3, *H* and *I*). Strikingly, the overexpression of FTO enhanced cellular resistance to RSL3-induced ferroptosis, with lower levels of ROS generation (Fig. 3*J*) and milder lipid peroxidation (Fig. 3*K*) compared with the control vector in CAL 27 and SCC-9 cells (Fig. 3*L*). Moreover, similar ferroptosis resistance role of FTO was observed in nontransformed human epithelial cells (HaCaT, Fig. S6). These results suggest a crucial role of FTO in cellular ferroptosis resistance.

Building on our findings that pharmacological inhibition of FTO demethylase activity by FB23-2 triggers ferroptosis, we examined whether FTO-mediated ferroptosis resistance depends on its catalytic function. We constructed an expression plasmid, encoding a catalytic-inactive mutant FTO, that has been reported to greatly compromise FTO demethylase activity (21) (Fig. S7, *A* and *B*). Flow cytometry results showed that while overexpression of wild-type FTO conferred cellular resistance to RSL3 induced ferroptosis, evidenced by reduced ROS accumulation and attenuated lipid peroxidation compared with empty vector controls, the catalytically inactive mutant FTO lost this protective capacity (Fig. S7, *C* and *D*). Collectively, these results suggested that the ferroptosis resistant role of FTO primarily depends on its demethylase activity.

### FTO promotes SRSF6 expression through its m6A demethylase function

**Figure 4:**
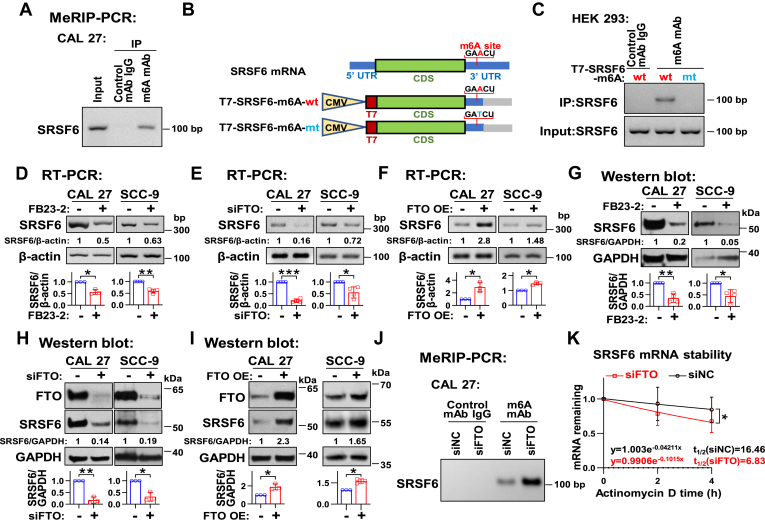
**FTO promotes the expression of SRSF6 through modulating RNA m6A modification.***A*, MeRIP-PCR analyzed the m6A modification on SRSF6 mRNA. *B*, the diagram of the predicted m6A site in SRSF6 mRNA 3′ UTR near the stop codon. The SRSF6 expression plasmids containing the wild-type (T7-SRSF6-m6A-wt) or mutant (T7-SRSF6-m6A-mt) m6A sites were constructed. *C*, MeRIP-PCR analyzed the m6A modification on mRNA transcripts of T7-SRSF6 in HEK 293 cells transduced with T7-SRSF6-m6A-wt or T7-SRSF6-m6A-mt plasmids. *D*–*I*, CAL 27 and SCC-9 cells were treated with FB23-2 and DMSO (*D* and *G*), transfected with siFTO and siNC (*E* and *H*), or transfected with FTO-FLAG and empty vector control plasmids (*F* and *I*). *D*–*F*, RT-PCR analyzed the mRNA expression of SRSF6, and β-actin served as a loading control. *G*–*I*, the protein expression of SRSF6 (*G*–*I*) and FTO (*H* and *I*) was analyzed by Western blot, and GAPDH served as a loading control. *J*, MeRIP-PCR analyzed the m6A modification on SRSF6 mRNA after cells transfected with siFTO and siNC. *K*, RT-qPCR analysis of the mRNA stability of SRSF6 in CAL 27 cells transfected with siNC or siFTO. β-actin served as a loading control. Data are means ± SD. ∗*p* < 0.05, ∗∗*p* < 0.01, ∗∗∗*p* < 0.001. DMSO, dimethyl sulfoxide; FTO, fat mass and obesity-associated protein; m6A, N6-methyladenosine; RT-qPCR, real-time quantitative reverse transcription PCR; RT-PCR, reverse transcription PCR; SRSF, serine and arginine rich splicing factor.

The m6A modification has been well described as the most abundant internal chemical mRNA modification in mammals. Therefore, we sought to investigate whether SRSF6 mRNA expression is regulated by m6A modification in an FTO-dependent manner. First, immunoprecipitation experiments were conducted using a monoclonal antibody against m6A. The results indicated that the anti-m6A antibody could bind to the mRNA fragments within the SRSF6 3′ UTR, specifically near the stop codon (Fig. 4*A*), where a potential m6A modification site with very high confidence was predicted *via* the SRMAP website. Subsequently, to confirm this m6A site, an SRSF6 expression plasmid containing this wild-type site (T7-SRSF6-m6A-wt) and a mutant SRSF6 expression plasmid carrying the mutated site (T7-SRSF6-m6A-mt) were constructed and transfected into cells (Fig. 4*B*). The methylated RNA immunoprecipitation PCR (MeRIP-PCR) results showed that the anti-m6A antibody could pull down the mRNA transcribed from “T7-SRSF6-m6A-wt” instead of “T7-SRSF6-m6A-mt” (Fig. 4*C*). These results imply that m6A modification is identified in SRSF6 mRNA at the site of “GAACU” near the stop codon.

Subsequently, we further explored whether the m6A modification on SRSF6 mRNA is regulated by FTO. Firstly, both the FTO demethylase inhibitor (FB23–2) treatment (Fig. 4*D*) and the silence of FTO by siRNA (Fig. 4*E*) significantly reduced the mRNA levels of SRSF6. Conversely, the overexpression of FTO increased the levels of SRSF6 mRNA (Fig. 4*F*). Consistently, both FB23-2 treatment (Fig. 4*G*) and FTO ablation (Fig. 4*H*) decreased SRSF6 protein expression, whereas FTO overexpression boosted it (Fig. 4*I*). Meanwhile, the catalytically inactive mutant FTO (Fig. S7*A*) lost the capacity of enhancing SRSF6 expression in CAL 27 and SCC-9 cells (Fig. S7*E*). These results indicated the promotion of FTO on SRSF6 expression in HNSC cells.

To further explore the effect of FTO protein on the m6A modification of SRSF6, we synthesized the RNA oligos containing the m6A modification at the site of “GAm6ACU” near the stop codon of SRSF6 mRNA previously shown in Figure 4*B*. And further *in vitro* m6A demethylation experiment showed that the m6A abundance of SRSF6 oligo RNA treated with FTO protein was obviously lower than that without treatment (Fig. S7*F*), suggesting that FTO protein may directly demethylate SRSF6 mRNA at the site of “GAm6ACU” near the stop codon. Moreover, MeRIP-PCR results showed that FTO knockdown enhanced the binding of the anti-m6A antibody to SRSF6 mRNA fragments containing the verified m6A-modified sequence “GAACU” near the stop codon (Fig. 4*J*). In addition, the actinomycin D experiment revealed that FTO silencing reduced the stability of SRSF6 mRNA (Fig. 4*K*). These results demonstrate that FTO promotes SRSF6 expression through its m6A demethylase activity.

### Depletion of SRSF6 or FTO inhibited the SREBP signaling pathway

**Figure 5:**
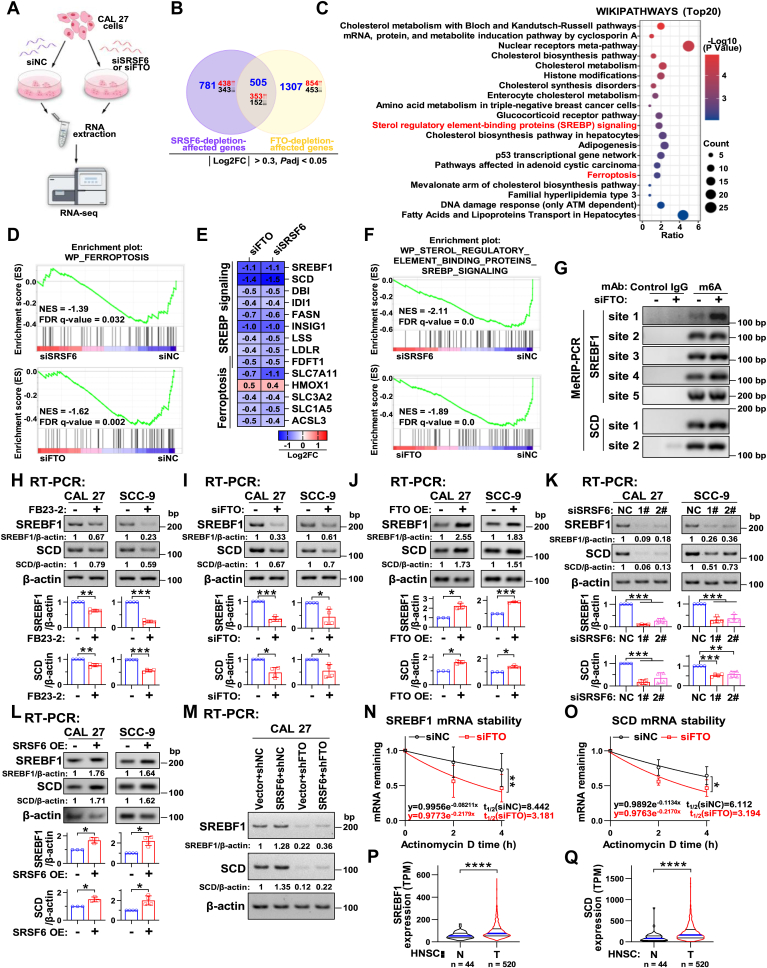
**FTO or SRSF6 silencing inhibits SREBF1/SCD expression.***A*–*F*, RNA sequencing (RNA-seq) and bioinformatic analysis in CAL 27 cells transfected with siFTO, siSRSF6-1#, and siNC. Three biological replicates were conducted per group. *A*, flow chart of the RNA-seq. *B*, the Venn diagram of the differentially expressed genes (DEGs) was created through the online tool EVenn. The thresholds of significant DEGs were | log 2 Fold Change (log 2 FC) | > 0.3 and *P*adj < 0.05. *C*, the WikiPathways (WP) enrichment analysis of the common downstream genes of SRSF6 and FTO analyzed by the Venn diagram in (*B*) was conducted using the online website DAVID. The top 20 signaling pathways (*p* < 0.05) were selected to draw the bubble diagram using the website Hiplot. *D*, the GSEA enrichment analysis of all genes in “WP-ferroptosis” pathways using the GSEA tool. *E*, heatmap of the log 2 FC values of genes in “WP-Sterol regulatory element-binding proteins (SREBP) signaling” and “WP-ferroptosis” pathways. *P*adj < 0.05. *F*, the GSEA enrichment analysis of all genes in “WP-Sterol regulatory element-binding proteins (SREBP) signaling” pathways using the GSEA tool. *G*, MeRIP-PCR analyzed the m6A modification on SREBF1 and SCD mRNA after CAL 27 cells transfected with siFTO and siNC. *H*–*L*, RT-PCR analysis of SREBF1 and SCD expression after cells treated with FB23-2 and DMSO (*H*), or transfected with siFTO and siNC (*I*), FTO-FLAG and control vector plasmids (*J*), siSRSF6 and siNC (*K*), as well as T7-SRSF6 and control vector plasmids (*L*). β-actin served as a loading control. Data are means ± SD, n = 3, 4, or 5. *M*, RT-PCR analysis of SREBF1 and SCD expression after CAL 27 cells stably cotransfected with T7-SRSF6 (empty vector as control) and shFTO (shNC as control) plasmids. β-actin served as a loading control. *N* and *O*, RT-qPCR analysis of the mRNA stability of SREBF1 (*N*) and SCD (*O*) in CAL 27 cells transfected with siNC or siFTO. β-actin served as a loading control. Data are means ± SD. *P* and *Q*, the analysis of mRNA levels of SREBF1 (*P*) and SCD (*Q*) in tumor tissues and normal tissues of HNSC from the TCGA database. The gene expression data were downloaded from the oncoDB website. ∗*p* < 0.05, ∗∗*p* < 0.01, ∗∗∗*p* < 0.001, ∗∗∗∗*p* < 0.0001. DMSO, dimethyl sulfoxide; FTO, fat mass and obesity-associated protein; GSEA, Gene Set Enrichment Analysis; HNSC, head and neck squamous cell carcinoma; m6A, N6-methyladenosine; RT-qPCR, real-time quantitative reverse transcription PCR; RT-PCR, reverse transcription PCR; SCD, stearoyl-CoA desaturase; SRSF, serine and arginine rich splicing factor; SREBF, sterol regulatory element binding transcription factor; TCGA, The Cancer Genome Atlas.

To further elucidate the underlying molecular mechanisms by which SRSF6/FTO regulate ferroptosis resistance and HNSC progression, we conducted RNA-Seq analysis of CAL 27 cells subjected to SRSF6 or FTO silencing (Fig. 5*A*). Firstly, we performed Venn diagram analysis based on the differentially expressed genes (DEGs, | log 2 Fold Change (log 2 FC) | > 0.3 and *P*adj < 0.05) from siSRSF6 *versus* siNC and siFTO *versus* siNC. The results indicated that 505 genes were common downstream genes of both SRSF6-depletion and FTO-depletion, including 353 upregulated and 152 downregulated genes (Fig. 5*B*). The WikiPathways enrichment analysis of these 505 genes revealed that the “Ferroptosis” pathway ranked among the top 20 most significant pathways (Fig. 5*C*). Meanwhile, Gene Set Enrichment Analysis (GSEA) analysis demonstrated that the ferroptosis-related pathway was also significantly underrepresented following the silencing of either SRSF6 (NES = −1.39, FDR q-value = 0.032, Fig. 5*D*) or FTO (NES = −1.62, FDR q-value = 0.002, Fig. 5*D*). Consistently, after the silencing of FTO or SRSF6 in CAL 27 cells, the mRNA expression levels of several reported antiferroptosis genes within the “Ferroptosis” pathway, including farnesyl-diphosphate farnesyltransferase 1 (FDFT1), SLC7A11, solute carrier family 3 member 2 (SLC3A2), solute carrier family 1 member 5 (SLC1A5), acyl-CoA synthetase long chain family member 3 (ACSL3), were significantly decreased, while the expression of the pro-ferroptosis gene heme oxygenase 1 (HMOX1) was increased (Fig. 5*E*).

Intriguingly, the top 20 most significant pathways also included the “Sterol regulatory element-binding proteins (SREBPs) signaling” pathway (Fig. 5*C*). SREBPs, also known as sterol regulatory element binding transcription factors (SREBFs), are transcription factors pivotal in the regulation of various lipid metabolism-related enzyme expression. Moreover, GSEA analysis indicated a significant downregulation of the gene set “WP_STEROL_REGULATORY_ELEMENT_BINDING_PROTEINS_SREBP_SIGNALING” in both siSRSF6 (NES = −2.11, FDR q-value = 0.0) and siFTO (NES = −1.89, FDR q-value = 0.0) groups compared with the siNC group (Fig. 5*F*). Consistently, SREBF1 (siSRSF6: log 2 FC = −1.1, *P*adj < 0.05; siFTO: log 2 FC = −1.1, *P*adj < 0.05) as well as its eight downstream target genes were significantly decreased following SRSF6 or FTO depletion (Fig. 5*E*). Notably, among these eight genes, stearoyl-CoA desaturase (SCD) exhibited the most significant change (siSRSF6: log 2 FC = −1.5, *P*adj < 0.05; siFTO: log 2 FC = −1.4, *P*adj < 0.05; Fig. 5*E*). SCD, acting as the key enzyme in catalyzing the conversion of saturated fatty acid into MUFA, has been reported to protect cells from ferroptosis (22). These results suggest that the SREBF1/SCD axis may represent a potential molecular mechanism by which SRSF6/FTO regulates ferroptosis resistance.

Subsequently, we sought to confirm the regulatory impact of SRSF6/FTO on SREBF1 and SCD. Both SREBF1 (23) and SCD (24) mRNAs have been reported to be modified by m6A. In addition, by predicting with the online tool SRMAP, we uncovered four potential m6A sites on SREBF1 mRNA with high confidence and one potential m6A site on SCD mRNA with very high confidence. The MeRIP-PCR results demonstrated that FTO knockdown enhanced the binding of anti-m6A antibody to the mRNA fragments containing these potential m6A sites (Fig. 5*G*). Also, both functional inhibition of FTO by FB23-2 (Fig. 5*H*) and FTO-depletion (Fig. 5*I*) significantly reduced the mRNA expression of SREBF1 as well as SCD in CAL 27 and SCC-9 cells. The overexpression of FTO caused significant increases in both SREBF1 and SCD expression at the transcriptional levels in HNSC cells (Fig. 5*J*). Moreover, the silence of SRSF6 induced the downregulation of SREBF1 and SCD mRNA (Fig. 5*K*), while the forced expression of SRSF6 promoted the expression of SREBF1 and SCD at mRNA level (Fig. 5*L*). To further confirm that SRSF6 promoted the mRNA expression of SREBF1 and SCD through the promotion of FTO protein expression, we conducted stable ablation of FTO in CAL 27 cells with stable SRSF6 overexpression. Consistent with the hypothesis, the results showed the inability of SRSF6 to fully rescue the reduction of SREBF1 and SCD mRNA expression induced by FTO depletion (Fig. 5*M*). Meanwhile, the actinomycin D assay indicated that FTO ablation diminished the mRNA stability of both SREBF1 (Fig. 5*N*) and SCD (Fig. 5*O*). In addition, our analysis of HNSC data from TCGA revealed that both SREBF1 (Fig. 5*P*) and SCD (Fig. 5*Q*) were overexpressed in tumor tissues. Collectively, these results suggest that the SRSF6/FTO axis promotes SREBF1 and SCD expression through FTO-mediated m6A demethylation.

### SRSF6/FTO enhance ferroptosis resistance through SREBF1/SCD axis

**Figure 6:**
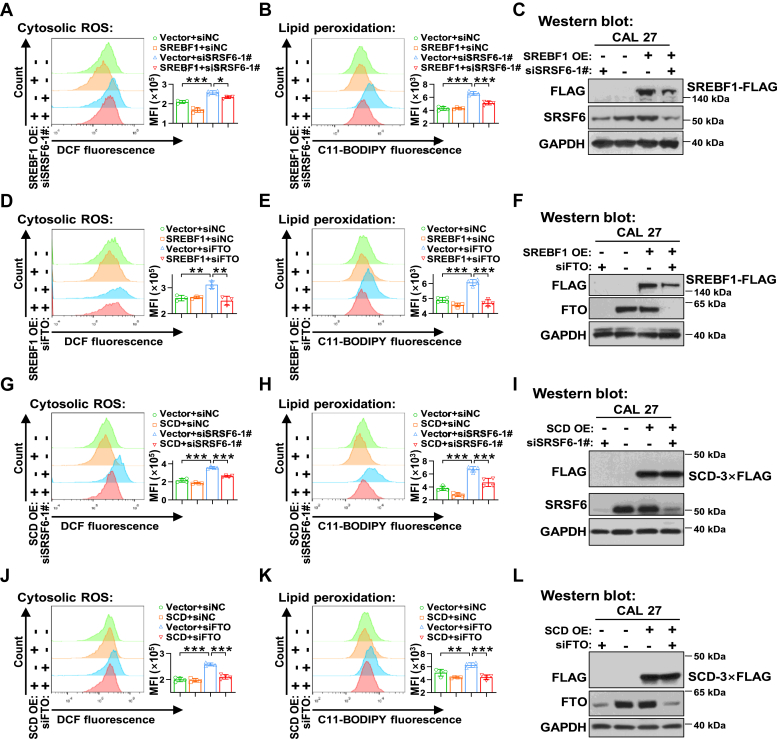
**Knockdown of SRSF6 or FTO induces ferroptosis through the inhibition of SREBF1/SCD.***A*–*F*, CAL 27 cells with or without SREBF1-FLAG overexpression were transfected with siSRSF6 (*A*–*C*) or siFTO (*D*–*F*). The cytosolic ROS level (*A* and *D*) and lipid peroxidation (*B* and *E*) were detected by flow cytometry. The overexpression of SREBF1-FLAG (*C* and *F*) and the knockdown efficiency of siSRSF6 (*C*) or siFTO (*F*) were analyzed by Western blot. GAPDH served as a loading control. *G*–*L*, Knockdown of SRSF6 (*G*–*I*) or FTO (*J*–*L*) was conducted by siRNAs in CAL 27 cells with or without SCD-3×FLAG overexpression. Flow cytometry was used to analyze the cytosolic ROS level (*G* and *J*) and lipid peroxidation (*H* and *K*). Western blot analyzed the overexpression of SCD-3×FLAG (*I* and *L*) and the silence efficiency of siSRSF6 (*I*) or siFTO (*L*), and GAPDH served as a loading control. Data are means ± SD, n = 4. ∗*p* < 0.05, ∗∗*p* < 0.01, ∗∗∗*p* < 0.001. FTO, fat mass and obesity-associated protein; ROS, reactive oxygen species; SCD, stearoyl-CoA desaturase; SREBF, sterol regulatory element binding transcription factor; SRSF, serine and arginine rich splicing factor.

SCD is a key enzyme in MUFA biosynthesis, a crucial cellular defense mechanism against ferroptosis (5). It has been reported that SREBF1 protects cells from ferroptosis through SCD activity (22). In the present study, the overexpression of SREBF1 substantially mitigated the overgeneration of ROS and cellular lipid peroxidation induced by the depletion of SRSF6 (Fig. 6, *A*–*C*) or FTO (Fig. 6, *D*–*F*) in CAL 27 cells. Also, after CAL 27 cells transfected with siSRSF6 (Fig. 6, *G*–*I*) or siFTO (Fig. 6, *J*–*L*), the overexpression of SCD showed lower levels of ROS and milder lipid peroxidation compared with vector control. These results suggest that both SRSF6 silencing and FTO ablation induce ferroptosis through the inhibition of SREBF1 and SCD in HNSC cells.

## Conclusions

**Figure 7:**
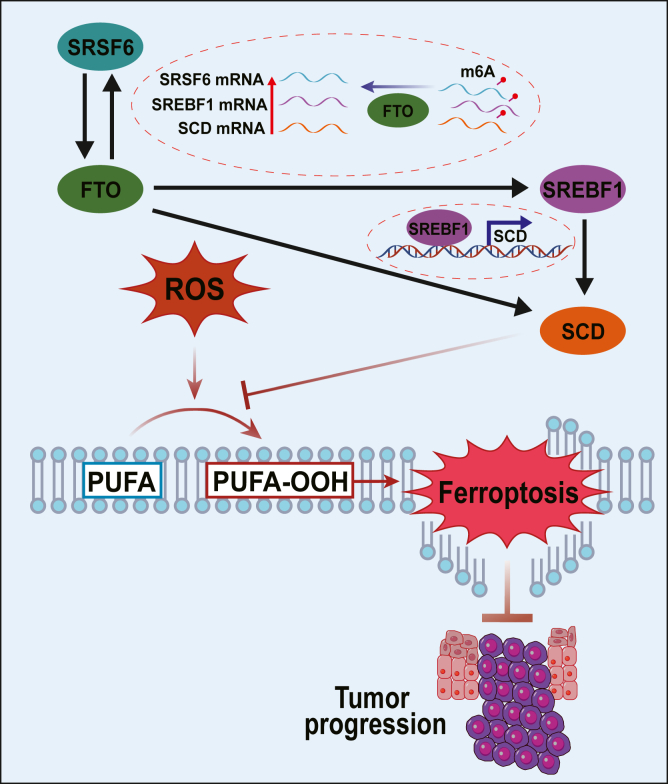
**Schematic model for the mechanism by which HNSC cells escape from ferroptosis through the SRSF6/FTO/SREBF1/SCD axis.** FTO, fat mass and obesity-associated protein; HNSC, head and neck squamous cell carcinoma; SCD, stearoyl-CoA desaturase; SREBF, sterol regulatory element binding transcription factor; SRSF, serine and arginine rich splicing factor.

In this study, we determined a critical oncogenic positive feedback loop containing SRSF6 and FTO in HNSC (Fig. 7). Both SRSF6 and FTO were essential for HNSC cell proliferation and promoted cellular resistance to ferroptosis. Mechanistically, SRSF6 promoted FTO protein expression. FTO promoted SRSF6 expression through exerting its RNA m6A demethylase activity, which increased the stability of SRSF6 mRNA. Furthermore, the SRSF6/FTO loop was identified to promote the expression of the SREBF1/SCD axis, which conferred cellular resistance to ferroptosis. Our study enriches the understanding of the molecular underpinnings of ferroptosis resistance and may pave the way for developing innovative strategies to overcome this resistance in HNSC and potentially other malignancies.

## Discussion

A growing number of studies have identified a significant increase in aberrant AS events in malignant cells, which is usually caused by aberrant expression or abnormal function of splicing factors. In this study, elevated mRNA expression of SRSF6 was identified in a variety of tumor tissues, including HNSC, cholangio carcinoma, colon adenocarcinoma, liver hepatocellular carcinoma, lung adenocarcinoma, lung squamous cell carcinoma, prostate adenocarcinoma, rectum adenocarcinoma, and stomach adenocarcinoma. SRSF6 has been reported to be overexpressed in several cancers such as lung cancers and colon cancers (12, 25). These results suggested a potential increase of SRSF6-related AS events in tumor cells. Moreover, we found a positive correlation between SRSF6 expression and Ki67 or PCNA expression at mRNA levels in HNSC, suggesting SRSF6 might be a candidate oncogene. Consistently, we found that SRSF6 is essential for HNSC cell proliferation. SRSF6 depletion significantly inhibited cell proliferation and colony formation in HNSC. Further research revealed that SRSF6 silencing induced ferroptosis, and SRSF6 overexpression promoted cellular ferroptosis resistance. Previous research has found that SRSF6-overexpressing mice developed pronounced epithelial hyperplasia in skin and small intestine tissues accompanied by elevated Ki67 expression (11). Furthermore, SRSF6 inhibited the expression of the most potent apoptotic isoform BimS by inhibiting the Bim exon 3 and exon 4 skipping in human neuroblastoma (26). Our study further revealed the effect of SRSF6 on the resistance of tumor cells to ferroptosis. These pieces of evidence suggest that SRSF6 acts as an oncogene required for cell proliferation and ferroptosis resistance.

Currently, the dysregulation of RNA modification and aberrant expression of related genes have been demonstrated to be involved in tumor occurrence and progression (27, 28, 29). As the most prevalent RNA modification in eukaryotic cells, m6A modification has been reported to regulate several RNA metabolic activities including mRNA splicing, RNA degradation, RNA stabilization, mRNA translation, and microRNA processing (30). Therefore, investigating the upstream regulatory mechanism of m6A-related genes can provide a better understanding of dysregulated RNA metabolism in tumors. In this study, we reported a novel mechanism regulating m6A RNA modification. Herein, SRSF6 was identified to decrease the m6A modification content of total RNA and promote the protein expression of FTO, an m6A demethylase, in HNSC cells. Previous studies have also found that STAT3 promoted the transcription of FTO in breast cancer cells (31). The circGPR137B was reported to act as a sponge for miR-4739 to upregulate its target FTO in hepatocellular carcinoma (32). In our study, we further determined that FTO mRNA expression was elevated in tumor tissues in HNSC from the TCGA database. The promotion of SRSF6 on FTO expression helps to explain the overexpression of FTO in tumors and other upstream regulatory pathways of FTO deserve further study.

FTO has been reported to exert oncogenic function in various cancers, including melanoma (33), breast cancer (15), acute myeloid leukemia (16), oral squamous cell carcinoma (34), and so on. In the present study, we found that FTO overexpression enhanced the ferroptosis resistance, while FTO silencing or inhibition induced obvious ferroptosis of tumor cells. Consistently, in colorectal cancers, FTO depletion induced ferroptosis through the suppression of glutathione peroxidase 4 (14, 35) and SLC7A11. In nasopharyngeal carcinoma, FTO enhanced radiotherapy resistance through repressing the radiation-induced ferroptosis (36). Apart from tumors, FTO has been reported to inhibit ferroptosis in doxorubicin-induced cardiotoxicity through the activation of P21/Nrf2 (37). Also, FTO overexpression can inhibit neuronal ferroptosis by suppressing Src-like protein-tyrosine kinase (FYN) expression and dynamin-related protein 1 (Drp1) activity in cerebral ischemia/reperfusion (I/R) injury (38). In addition, the apoptosis induced by FTO ablation has been reported in several cancer types including breast cancer (15), colorectal cancer (39), acute myeloid leukemia (20), and so on. These results suggested the potential oncogenic roles of FTO in tumor cells by resisting cell ferroptosis and cell apoptosis.

In the present study, it was determined that the SRSF6/FTO loop promotes cellular resistance to ferroptosis. Knockdown of both SRSF6 and FTO caused a remarkable accumulation of ROS and lipid peroxidation. Ferroptosis has been recognized as a promising target in cancer treatment, and several ferroptosis inducers displayed antitumor effects, which might provide new strategies for overcoming or limiting the resistance of tumors to traditional chemotherapeutic drugs. However, tumors developed defense systems against ferroptosis, which complicated treatment options. It is currently believed that the restriction of PUFA-PL synthesis by MUFAs is one of the key reinforcements of cellular ferroptosis resistance in cancer cells (5). SCD is a key enzyme catalyzing the critical committed step in the biosynthesis of MUFA. In the present study, we determined that SRSF6/FTO promoted the expression of SREBF1 and SCD in HNSC cells. Meanwhile, the overexpression of both SREBF1 and SCD effectively limited ferroptosis induced by SRSF6 depletion or FTO depletion. Thus, targeting the upstream SRSF6/FTO feedback loop might be an alternative strategy to overcome cellular ferroptosis resistance in cancer therapy.

## Experimental procedures

### Cells and reagents

Human HNSC cell line CAL 27 and human embryonic kidney (HEK) 293 cell line were grown in Dulbecco’s modified Eagle medium (DMEM; HyClone) supplemented with 10% fetal bovine serum (FBS; Gibco) and 1% antibiotic–antimycotic (Gibco). Human HNSC cell line SCC-9 was cultured in DMEM: F12 (HyClone) supplemented with 10% FBS, 400 ng/ml hydrocortisone, and 1% antibiotic–antimycotic. Human nontransformed epithelial cell HaCaT cell line was cultured in minimum essential medium (MEM; Procell Life Sciences) supplemented with 10% FBS and 1% antibiotic–antimycotic. CAL 27, SCC-9, and HEK 293 cells were obtained and authenticated as previously described (40). HaCaT cells were purchased from and authenticated by Procell Life Sciences company. All cells were tested negative for *mycoplasma* contamination.

The FTO inhibitor (FB23–2) and the transcription inhibitor (actinomycin D) were purchased from Selleck Chemical Company. The ferroptosis inhibitor (Fer-1) and agonist (RSL3) were bought from MedChemExpress.

### Plasmids, siRNAs, and shRNAs

The human SRSF6 complementary DNA (cDNA) was amplified from CAL 27 cells using the primers 5′ GTCCGCCGTTCGACAACCAG 3′ and 5′ TTCCATAATAATGTGCAAACAAGGAG 3′. A T7 tag was added to the 5′ end of the SRSF6 ORF. The T7-SRSF6 fusion fragment was cloned into pLVX-IRES-Puro (Clontech) to generate SRSF6 expression plasmid.

To further confirm the m6A modification site in SRSF6 mRNA, we cloned the 3′ UTR sequence (T7-SRSF6-m6A-wt) containing the predicted m6A modification motif (“GAACT”) near the stop codon and performed point mutation (“GATCT”, T7-SRSF6-m6A-mt). T7-SRSF6-m6A-wt was cloned from T7-SRSF6 plasmid using the following primers: 5′ GGATCTATTTCCGGTGAATTCGGGATCTTGGTGGCGTGA 3′ and 5′ CGCGGCCGCTCTAGAACTAGTGTAGGAAAGTGTTCCATAATAATGTGCAAACAAGGAGTTCTGAGTTAATCTCTGGAACTCGACCTGGA 3′. T7-SRSF6-m6A-mt was cloned from T7-SRSF6 plasmid using the following primers: 5′ GGATCTATTTCCGGTGAATTCGGGATCTTGGTGGCGTGA 3′ and 5′ CGCGGCCGCTCTAGAACTAGTGTAGGAAAGTGTTCCATAATAATGTGCAAACAAGGAGATCTGAGTTAATCTCTGGAACTCGACCTGGA 3′. Both plasmids were cloned into pLVX-IRES-Puro plasmid at EcoRI and SpeI sites.

For the transient overexpression of human FTO, the pCMV3-FTO-Flag (#HG12125-CF) plasmid was purchased from Sino Biological Inc. For the stable overexpression, FTO-Flag was amplified from the pCMV3-FTO-Flag plasmid and cloned into pLVX-IRES-ZSgreen1 plasmid at XhoI and NotI sites. The catalytically inactive mutant of FTO plasmid was generated by overlapping PCR with the following primers: 5′ TGAGCTGGCATCATGATCCGAATCTGGTGGACAGGTCAGC 3′ and 5′ GCTGACCTGTCCACCAGATTCGGATCATGATGCCAGCTCA 3′.

The long or short 3′ UTR of FTO was amplified from the cDNA of CAL 27 cells using the following primers: long 3′ UTR with 5′ AAGGAGCACAAGTCTCAGGCG 3′ and 5′ TGATTTCAATTTTTTTATTTTTAATTTTTGTGGGTACATAGGAGGCGTAT 3′, short 3′ UTR with 5′ AAGGAGCACAAGTCTCAGGCG 3′ and 5′ TTTCTTATCTCATCCTTTTAATATGTTTATTG 3′. Subsequently, the two fragments were inserted into the pEGFP-C1 plasmid downstream the GFP gene by seamless cloning.

Human SREBF1-FLAG was amplified from the pCMV3-SREBF1-FLAG plasmid (#HG17512, Sino Biological Inc) and inserted into pLVX-IRES-Puro plasmid by seamless cloning (Vazyme Biotech).

For the overexpression of human SCD, the ORF was cloned from the cDNA of CAL 27 cells using the primers 5′ CAAGATGCCGGCCCACTTGC 3′ and 5′ CTCAGCCACTCTTGTAGTTTCCATC 3′. Subsequently, a C-terminal 3×FLAG tag, containing a stop codon at the 3′ end, was cloned after the SCD ORF. Then the SCD-3 × FLAG fusion fragment was inserted into pLVX-IRES-puro by seamless cloning.

The sequences of siRNA and shRNA are provided in Table S1.

### Transfection

For the stable transfection of gene expression plasmids and shRNA plasmids in CAL 27 and SCC-9 cells, a lentiviral expression system was used. The plasmids were cotransfected with psPAX2 and pMD2. G into HEK 293T cells to generate lentiviral particles using Lipofectamine 2000 (Invitrogen) according to the manufacturer’s protocol. Two days after transfection, the virus-containing supernatant was collected and filtered through a 0.45 μm filter. Then the filtered supernatant was added to cells pretreated with 8 μg/ml polybrene (Santa Cruz Biotechnology).

The transient transfection of plasmids and siRNAs was conducted using Lipofectamine 3000 (Invitrogen) in accordance with the manufacturer’s protocol.

### RNA purification, semiquantitative reverse transcription PCR, and real-time quantitative reverse transcription PCR

Total RNA was purified using the Total RNA Miniprep Kit (Axygen). To eliminate genomic DNA contamination, total RNA was treated with DNase I (#M1682, Thermo Fisher Scientific). Next, the reverse transcription reaction was carried out using the Maxima H Minus cDNA Synthesis Master Mix (#M1682, Thermo Fisher Scientific). Subsequently, cDNA was then analyzed through semi-quantitative PCR (#P131, Vazyme Biotech) or quantitative real-time PCR (#A57156, Thermo Fisher Scientific) following the manufacturer’s protocol. The primers used are listed in Table S2.

### Western blot

Protein samples were collected using 2 × SDS sample buffer and then denatured for 3 min at 95 °C. Then the protein samples were separated in 4 to 12% PAGE gels (GeneScript) or 10% PAGE gels (Vazyme Biotech) and transferred to nitrocellulose membranes (Pall Corporation). After blocking with 5% skim milk, the membrane was probed with different specific primary antibodies as follows: rabbit anti-SRSF6 antibody (#ab140623, 1:2000, Abcam), rabbit anti-FTO antibody (#27226, 1:2000, Proteintech), rabbit anti-Flag antibody (#20543, 1:2000, Proteintech), mouse anti-GAPDH antibody (#sc-47724, 1:1000, Santa Cruz Biotechnology).

### Cellular ROS and lipid peroxidation measurement

The cellular ROS was detected using the reactive oxygen species assay kit (Beyotime). In short, cells were collected and resuspended in DMEM with 10 μM 2′,7′-dichlorodihydrofluorescein diacetate and were incubated at 37 °C for 20 min. The fluorescein isothiocyanate channel of the flow cytometer was used to detect the fluorescence intensity of DCF at an excitation wavelength of 488 nm.

The BODIPY 581/591 C11 probe (Thermo Fisher Scientific), the fluorescence excitation and emission of which shifted from 581/591 nm to 490/510 nm after oxidation, was used to measure lipid peroxidation. Briefly, cells were collected and resuspended in PBS with 5 μM C11 BODIPY dye and were incubated at 37 °C for 30 min. Subsequently, the fluorescence intensity of cells was analyzed by flow cytometer using channel fluorescein isothiocyanate.

FlowJo software was utilized to analyze the flow cytometry data.

### RNA immunoprecipitation

RIP assay was performed using a Dynabeads Protein A immunoprecipitation kit (Thermo Fisher Scientific). Briefly, cells stably overexpressing T7-SRSF6 were treated with UV irradiation and lysed with cellular lysis buffer (Thermo Fisher Scientific). Subsequently, total cell lysates were incubated with protein A beads bound with anti-T7 antibody (#13246, CST) overnight with rotation at 4 °C. Subsequently, 2 × SDS sample buffer and anhydrous ethanol with the help of glycogen (Thermo Fisher Scientific) were used to collect the immunoprecipitated protein and RNA, followed by the Western blot and reverse transcription PCR analysis, respectively.

### Subcutaneous xenograft model

Female 8-week-old Balb/c nude mice were used for the xenograft experiments in this study. All mice were housed in a specific-pathogen-free environment at Wuhan University. CAL 27 cells, stably transfected using lentivirus, were injected into both sides of the dorsum of nude mice subcutaneously. Tumor growth was monitored by measuring and calculating tumor volume using a vernier caliper according to the following formula: tumor volume = length × width^2^ × π/6. Mice were sacrificed on day 47 after tumor inoculation. Next, tumors were dissected, collected, and weighed. All animal experiments were conducted under guidelines approved by the institutional Animal Ethics Committee, Hospital of Stomatology, Wuhan University (protocol code S07921060G).

### RNA sequencing (RNA-seq) analysis

Total RNA was extracted from CAL 27 cells transfected with siRNAs using the PureLink RNA Mini Kit (Invitrogen). The RNA-seq experiments were performed by Wuhan Ruixing Biotechnology Co. Ltd using the Illumina Novaseq 6000 system. Differential gene expression was analyzed using the DESeq2 software. Then the pathway enrichment of the differentially expressed genes and all genes were analyzed using the DAVID database and the GSEA tool, respectively.

### MeRIP

MeRIP assay was performed according to the reported protocol (41). After being treated with DNase I (#M1682, Thermo Fisher Scientific) to eliminate genomic DNA contamination, total RNA was precipitated overnight with sodium acetate (Invitrogen) and glycogen (Thermo Fisher Scientific) and resuspended in RNase-free water (Thermo Fisher Scientific). Subsequently, the treated RNA was fragmented using ZnCl_2_. Next, the prewashed Pierce Protein A Magnetic Beads (Thermo Fisher Scientific) were incubated with m6A antibody (#ab190886, Abcam) or IgG (#ab172730, Abcam) for 2 h. Then the purified fragmented RNA and magnetic beads were mixed in the wash buffer according to the protocol. After digested by proteinase K, RNA was treated with sodium acetate and glycogen for precipitation at −80 °C overnight.

### Colony formation assay

CAL 27 and SCC-9 cells were plated at 2000 cells per well in six-well plates and cultured for 2 weeks. Subsequently, the cells were fixed with 4% paraformaldehyde and stained with crystal violet. Cell clusters with more than 50 cells were counted as clones.

### Dot blot assay for m6A

Total RNA samples were denatured at 65 °C for 5 min. Subsequently, RNA samples were spotted onto the nitrocellulose membranes (Pall Corporation). After conducting UV-crosslinking, the membranes were stained with methyl blue and photographed. Next, the membranes were blocked with 5% skim milk and incubated with rabbit anti-m6A antibody (#ab190886, Abcam). After incubation with a secondary antibody (#ab97051, Abcam), the membrane was detected with the enhanced chemiluminescence (ECL) system.

### *In vitro* demethylation assay

For *in vitro* demethylation assays, FTO protein was bought from Beyotime company and the m6A modified RNA oligo, with the sequence of “CAGAm6ACUCC”, was synthesized by Generay Biotechnology. FTO protein and SRSF6 m6A modified RNA oligo were incubated at 25 °C for 2 h with rotation. Subsequently, 5 mM EDTA was added to the reaction mixture followed by the incubation at 95 °C for 5 min to terminate the reaction. Finally, the RNA mixture was conducted the dot blot assay to detect the RNA m6A modification level.

### RNA stability assay

After transfected with siRNAs, cells were treated with actinomycin D. Subsequently, total RNA samples were collected at 0, 2, and 4 h. The expression of interested mRNA was analyzed using real-time quantitative reverse transcription PCR. The degradation curve and half-life of mRNA were analyzed using GraphPad Prism software.

### Statistical analysis

The band intensity of gels was analyzed by ImageJ. The individual data points in the bar graphs represents “n” independent biological replicates, as indicated in the figure legends. The differences in the mean values between the two groups were analyzed using the two-tailed *t* test, while the differences among multiple groups were analyzed using a one-way ANOVA test in the GraphPad Prism software. After the square root transformation of the values, differences of tumor volume and tumor weight between groups were analyzed using a one-way ANOVA corrected for two-stage linear step-up procedure of Benjamini, Krieger, and Yekutieli.

## Data availability

The gene expression and clinical data in the TCGA database analyzed in this study can be accessed on the oncoDB website. Sequencing reads are available in the Genome Sequence Archive in National Genomics Data Center, China National Center for Bioinformation/Beijing Institute of Genomics, Chinese Academy of Sciences (GSA-Human: HRA008470) that are publicly accessible at https://ngdc.cncb.ac.cn/gsa-human. All other data needed to evaluate the conclusions in the article are present in the paper and/or the Supplementary Materials.

## Supporting information

This article contains supporting information.

## Ethics approval

All animal experiments were conducted under guidelines approved by the institutional Animal Ethics Committee, Hospital of Stomatology, Wuhan University (protocol code S07921060G).

## Conflict of interest

The authors declare that they have no conflicts of interest with the contents of this article.
