# Supplementary material for: The splicing factor SRSF6 mediates ferroptosis resistance in head and neck squamous cell carcinoma through induction of stearoyl-CoA desaturase
Source: J Biol Chem. 2025 Jul 23;301(9):110509. doi: 10.1016/j.jbc.2025.110509 (PMC12390944; doi:10.1016/j.jbc.2025.110509)
Supplement: Supplementary Figure [file mmc1.pdf]

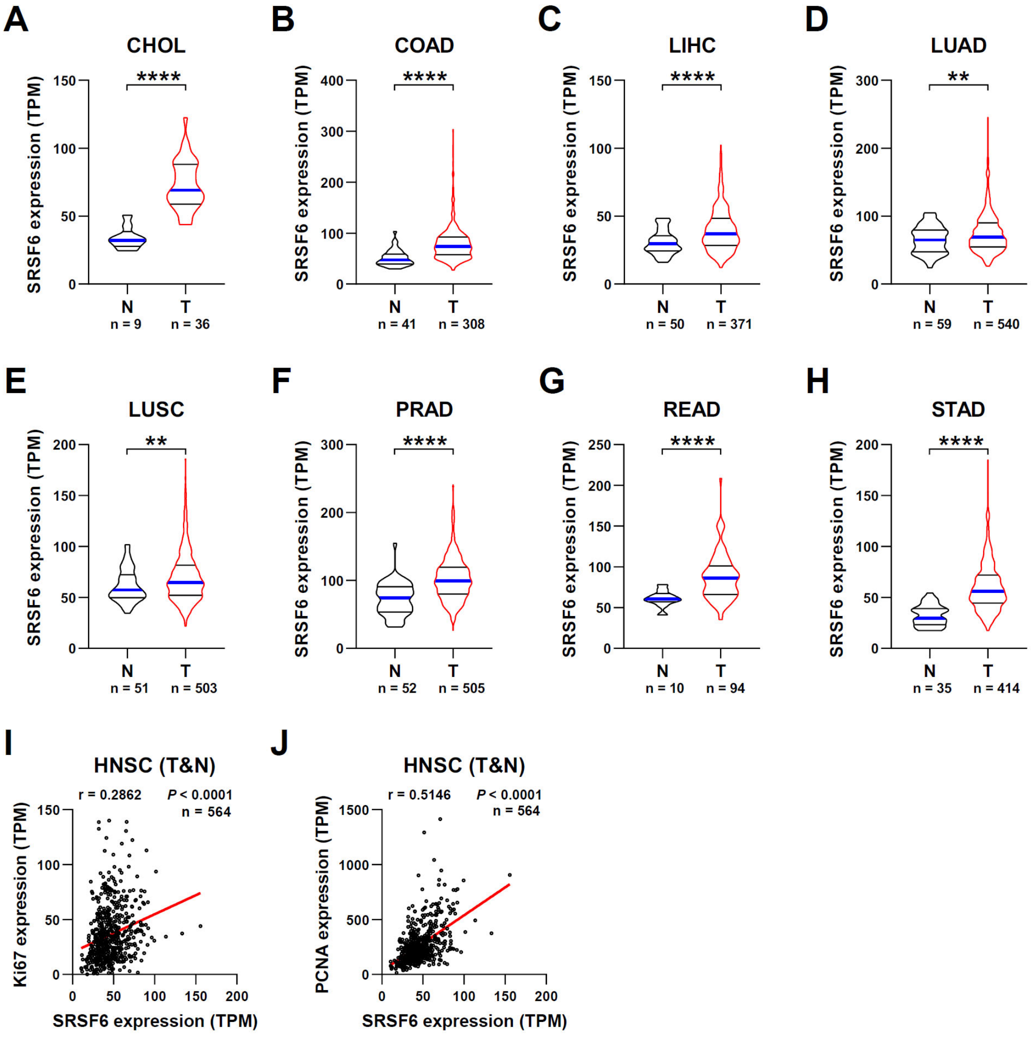

Figure S1. Overexpressed SRSF6 in tumor. (A to H) The statistical analysis of the transcriptional level of SRSF6 in tumor tissues and normal tissues from the TCGA database. **(I and J)** Spearman correlation between the transcriptional levels of SRSF6 and Ki67 (I) or PCNA (J) in HNSC (including tumor tissues and normal tissues) from the TCGA database. The gene expression data were downloaded from the oncoDB website. $^{**}P < 0.01$, $^{****}P < 0.0001$. CHOL, cholangio carcinoma; COAD, colon adenocarcinoma; LIHC, liver hepatocellular carcinoma; LUAD, lung adenocarcinoma; LUSC, lung squamous cell carcinoma; PRAD, prostate adenocarcinoma; READ, rectum adenocarcinoma; STAD, stomach adenocarcinoma; HNSC, head and neck squamous cell carcinoma.

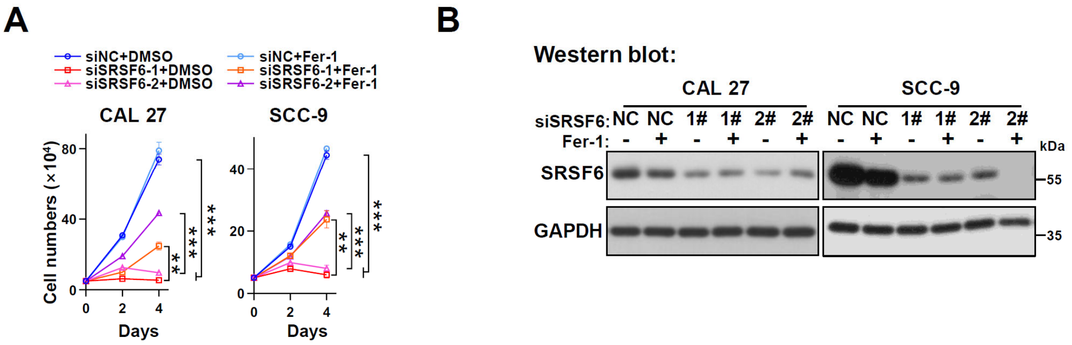

Figure S2. Ferroptosis inhibitor Fer-1 rescues proliferation inhibition induced by siSRSF6. After transfected with siSRSF6 or siNC, CAL 27 and SCC-9 cells were treated with the antioxidant ferrostain-1 (Fer-1) or DMSO. **(A)** Proliferation curves of HNSC cells (CAL 27 and SCC-9). Data are means ± SD, n = 4. **$P < 0.01$, ***$P < 0.001$. **(B)** Western blot analyzed the knockdown efficiency of siSRSF6 and GAPDH served as a loading control.

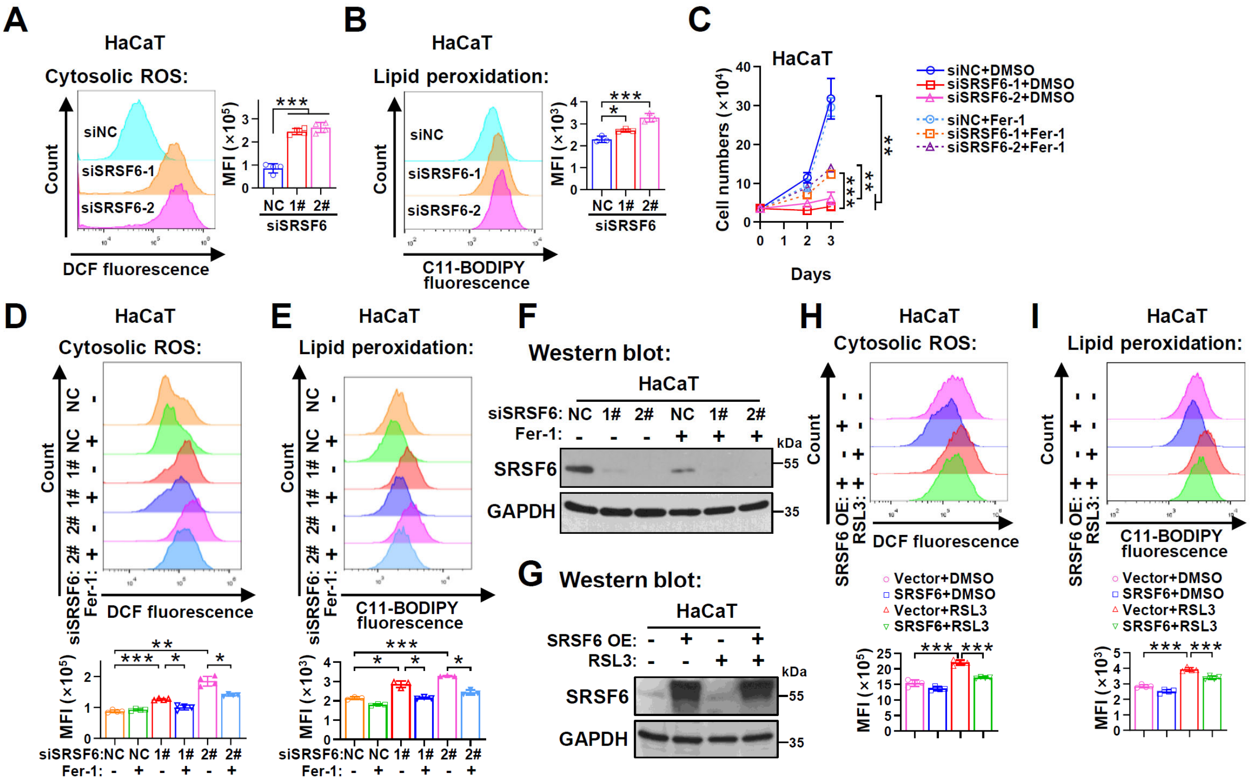

Figure S3. SRSF6 promotes ferroptosis resistance in non-transformed epithelial cells. (A and B) HaCaT cells were transfected with siRNAs targeting SRSF6 (siSRSF6-1#, siSRSF6-2#) or siNC. Then flow cytometry was used to detect the levels of ROS (A) and lipid peroxidation (B). **(C-F)** After transfected with siSRSF6 or siNC, HaCaT cells were treated with Fer-1 or DMSO. **(C)** Proliferation curves of HaCaT cells. **(D and E)** Flow cytometry analysis of the levels of ROS (D) and lipid peroxidation (E). **(F)** Knockdown efficiency of siSRSF6 in HaCaT cells was confirmed by Western blot, and GAPDH served as a loading control. **(G-I)** HaCaT cells stably overexpressing T7-SRSF6 or empty control vector were treated with RSL3 or DMSO. **(G)** Western blot analysis of the overexpression of T7-SRSF6, and GAPDH served as a loading control. **(H and I)** The levels of ROS (H) and lipid peroxidation (I) were analyzed by flow cytometry. Data are means ± SD, n = 3 or 4. *$P < 0.05$, **$P < 0.01$, ***$P < 0.001$.

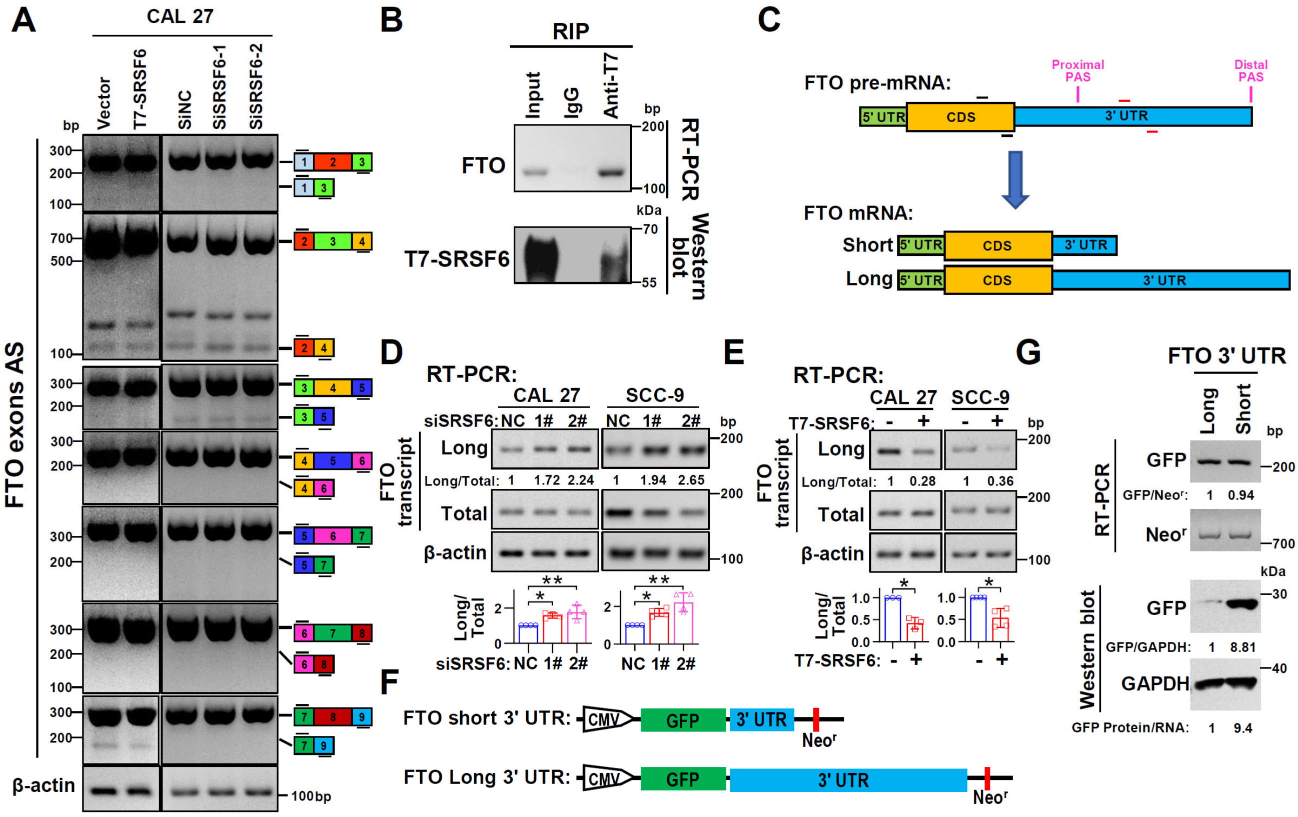

Figure S4. SRSF6 promotes the selection of the proximal PAS of FTO. (A) RT-PCR analysis of the exons 2-8 skipping of FTO mRNA after CAL 27 cells transfected with T7-SRSF6 or empty control vector plasmids as well as transfected with siSRSF6 or siNC. Diagram on the right showed the splice products of FTO mRNA. The short lines represent primers. **(B)** The interaction between SRSF6 protein and FTO mRNA were analyzed by RNA immunoprecipitation (RIP) using anti-T7 antibody in cells overexpressing T7-SRSF6. Immunoprecipitated RNA of FTO was analyzed by RT-PCR and immunoprecipitated T7-SRSF6 protein was confirmed by Western blot. **(C)** Diagram of FTO mRNA, the proximal polyadenylation signal (PAS) and distal PAS. The short black and red lines represent the primers detecting the total and long transcripts of FTO mRNA, respectively. **(D and E)** RT-PCR analysis of the levels of the long and total transcripts of FTO mRNA after cells transfected with siSRSF6 or siNC (D) as well as transfected with T7-SRSF6 or empty vector control plasmids (E). β-actin served as a loading control. The histograms below summarize the relative ratio of the long vs. total transcript of FTO mRNA. Data are means ± SD, n = 3 or 4. **P* < 0.05, ***P* < 0.01. **(F)** Schematic diagram of the plasmids containing the long or short FTO 3' UTR. **(G)** After transfecting HEK 293 cells with the FTO long or short 3' UTR plasmids in (F), the relative mRNA and protein level of GFP were analyzed by RT-PCR and Western blot, respectively. Neo^r gene in the vector plasmid and GAPDH were served as the loading control of RT-PCR and Western blot, respectively.

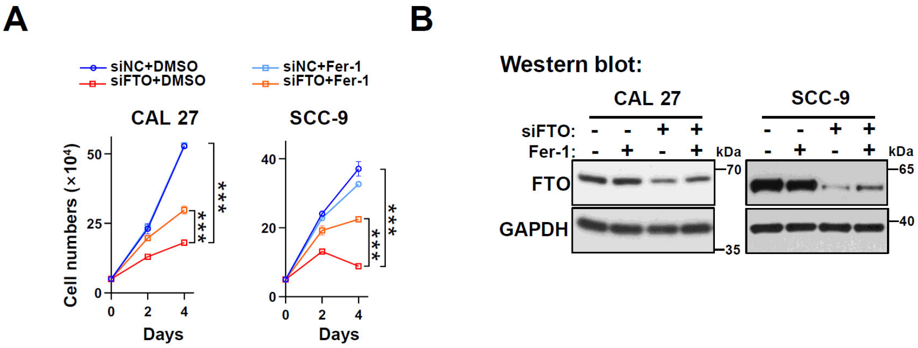

Figure S5. Fer-1 rescues proliferation inhibition induced by siFTO. After transfected with siFTO or siNC, HNSC cells (CAL 27 and SCC-9) were treated with the ferroptosis inhibitor (Fer-1) or DMSO. **(A)** Proliferation curves of CAL 27 and SCC-9. Data are means ± SD, n = 4. ***$P < 0.001$. **(B)** Knockdown efficiency of siFTO was analyzed by Western blot and GAPDH served as a loading control.

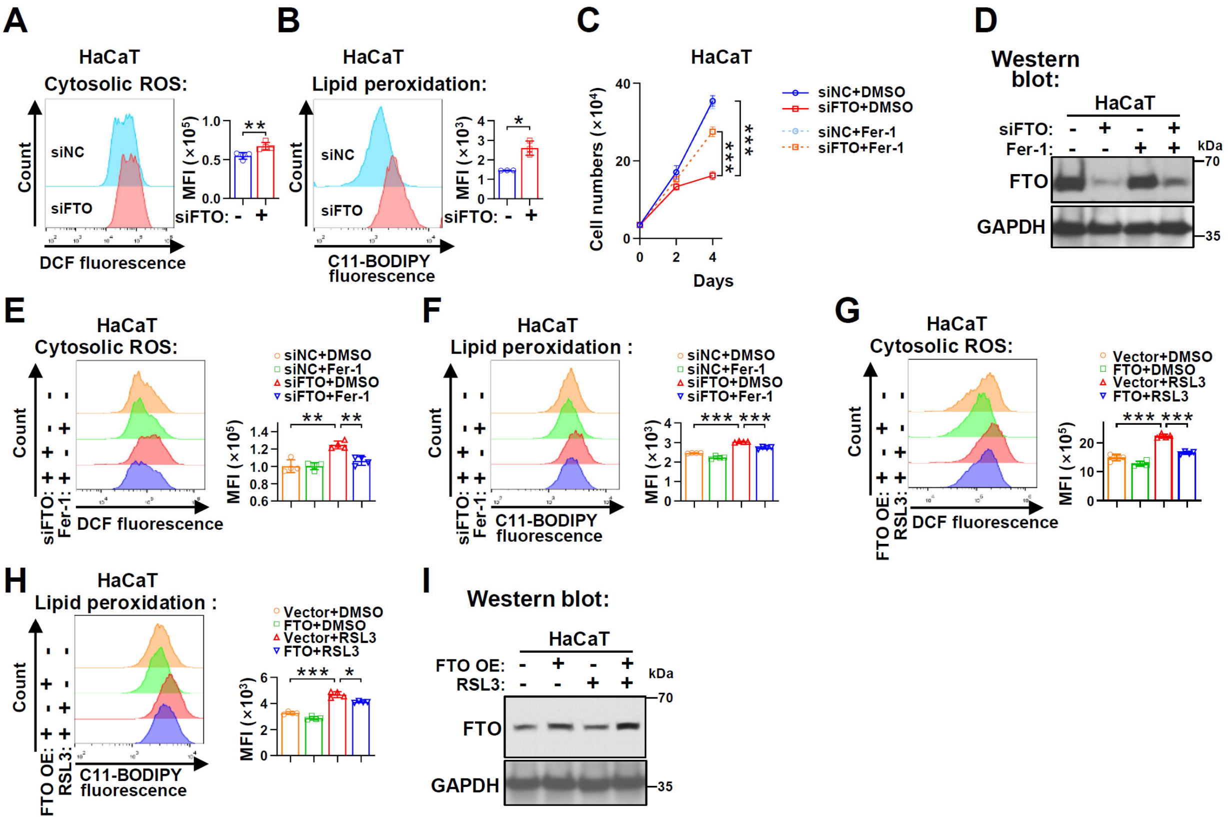

Figure S6. FTO promotes ferroptosis resistance in HaCaT cells. (A and B) HaCaT cells were transfected with siFTO or siNC. The cytosolic ROS (A) and lipid peroxidation (B) were analyzed by flow cytometry. **(C-F)** After transfected with siFTO or siNC, HaCaT cells were treated with ferroptosis inhibitor Fer-1 or DMSO. **(C)** Proliferation curves of HaCaT cells. **(D)** Western blot analyzed the knockdown efficiency of siFTO. GAPDH served as a loading control. **(E and F)** Flow cytometry analysis of ROS (E) and lipid peroxidation (F). **(G-I)** HaCaT with or without FTO-FLAG overexpression were treated with DMSO or ferroptosis inducer RSL3. **(G and H)** Flow cytometry analysis of ROS (G) and lipid peroxidation (H). **(I)** Western blot analysis of the overexpression of FTO. GAPDH served as a loading control. Data are means ± SD, n = 3 or 4. *$P < 0.05$, **$P < 0.01$, ***$P < 0.001$.

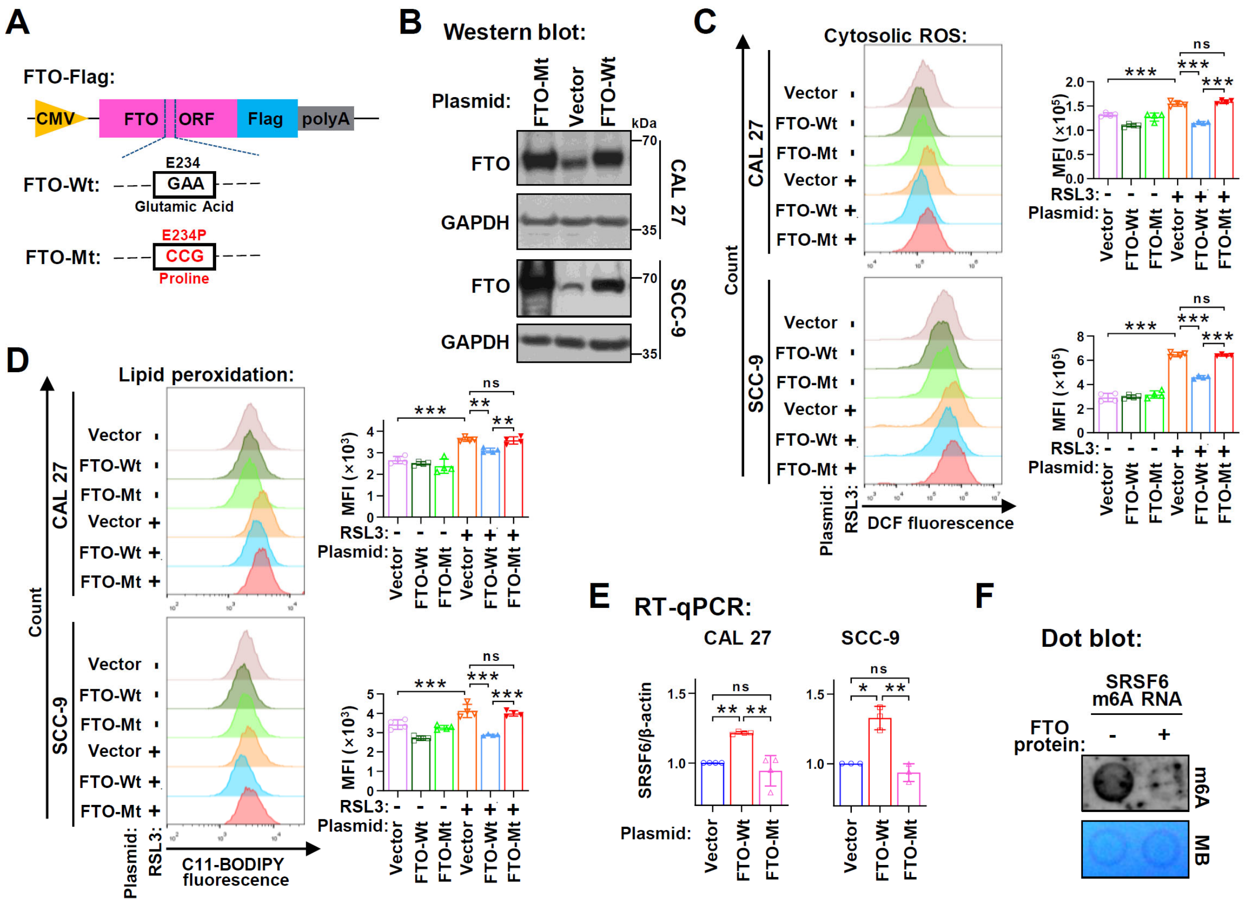

Figure S7. The enhancement of FTO on ferroptosis resistance and SRSF6 expression relies on its demethylase activity. (A) Diagram of the expression plasmid encoding a catalytic-inactive mutant FTO (FTO-Mt) as well as the wild-type FTO (FTO-Wt). **(B)** CAL 27 and SCC-9 cells were transfected with the plasmids, including FTO-Wt, FTO-Mt, or empty vector control. Western blot was used to verify the overexpression efficiency of FTO, and GAPDH served as a loading control. **(C and D)** Cells in (B) were treated with RSL3. Flow cytometry analyzed the levels of ROS (C) and lipid peroxidation (D). **(E)** RT-qPCR analyzed the mRNA expression of SRSF6 after cells transfected with FTO-Mt, FTO-Wt, or empty control vector plasmids. β-actin served as a loading control. **(F)** The in vitro m6A demethylation assay followed by the RNA m6A dot blot assay. FTO protein was incubated with m6A modified SRSF6 RNA oligos, with the sequence of "CAGAm6ACUCC". Subsequently, dot blot assay for m6A was conducted to detect the abundance of RNA m6A modification. Methylene blue (MB) staining served as a loading control. Data are means ± SD. *$P < 0.05$, **$P < 0.01$, ***$P < 0.001$.
